# Carminic acid supplementation protects against fructose-induced kidney injury mainly through suppressing inflammation and oxidative stress via improving Nrf-2 signaling

**DOI:** 10.18632/aging.202794

**Published:** 2021-04-04

**Authors:** Qiang Li, Qifei Xu, Jun Tan, Linfeng Hu, Chenxu Ge, Minxuan Xu

**Affiliations:** 1 Chongqing Key Laboratory of Medicinal Resources in the Three Gorges Reservoir Region, School of Biological and Chemical Engineering, Chongqing University of Education, Chongqing 400067, PR China; 2 Research Center of Brain Intellectual Promotion and Development for Children Aged 0-6 Years, Chongqing University of Education, Chongqing 400067, PR China; 3 Key Laboratory of Biorheological Science and Technology (Chongqing University), Ministry of Education, College of Bioengineering, Chongqing University, Chongqing 400030, PR China; 4 Department of Radiology, Linyi People Hospital, Linyi 276000, PR China

**Keywords:** kidney injury, carminic acid (CA), inflammation, oxidative stress, Nrf-2

## Abstract

Excessive fructose (Fru) intake has become an increased risk for chronic kidney disease progression. Despite extensive researches that have been performed to develop effective treatments against Fru-induced renal injury, the outcome has achieved limited success. In this study, we attempted to explore whether carminic acid (CA) could influence the progression of Fru-induced kidney injury, and the underlying molecular mechanism. At first, our *in vitro* results showed that CA significantly reduced inflammation in mouse tubular epithelial cells and human tubule epithelial cells stimulated by Fru. The anti-inflammatory effects of CA were associated with the blockage of nuclear factor-κB (NF-κB) signaling. In addition, Fru-exposed cells showed higher oxidative stress, which was effectively restrained by CA treatment through improving nuclear factor (erythroid-derived 2)-like 2 (Nrf-2) nuclear translocation. Importantly, we found that Fru-induced inflammation and oxidative stress were accelerated in cells with Nrf-2 knockdown. What’s more, in Fru-stimulated cells, CA-alleviated inflammatory response and reactive oxygen species (ROS) production were evidently abolished by Nrf-2 knockdown. The *in vivo* analysis demonstrated that Fru led to metabolic disorder, excessive albuminuria and histologic changes in renal tissues, which were effectively reversed by CA supplementation. We confirmed that CA significantly reduced inflammation and oxidative stress in the kidneys of mice through regulating NF-κB and Nrf-2 signaling pathways, eventually alleviating the progression of chronic kidney injury. Taken together, these results identified CA as a potential therapeutic strategy for metabolic stress-induced renal injury through restraining inflammation and oxidative stress via the improvement of Nrf-2 signaling.

## INTRODUCTION

Excessive fructose (Fru) intake has become an increased risk factor for kidney diseases in animals and humans, associated with high mortality [1, 2]. In recent decades, Fru consumption, mainly as table sugar or high fructose corn syrup, has been involved in metabolic complications, including insulin resistance and metabolic disorders [3, 4]. Additionally, Fru as a highly lipogenic monosaccharide can result in insulin signaling abnormality, dyslipidemia, abnormal glucose metabolism, liver steatosis and renal dysfunctions [5–7]. Fru consumption leads to glomerular podocyte dysfunction and greater albuminuria, contributing to the development of chronic kidney disease [8, 9]. Although great progress has been made, Fru-induced renal injury still cannot be effectively treated. Therefore, it is urgently necessary to develop more effective medicines to prevent or slow down its progression [10, 11].

Increasing studies have demonstrated that inflammatory response contributes to Fru-induced kidney disease in rodent animals [8, 12, 13]. Metabolic stresses including Fru induce inflammation, disrupting cell function and pathologic changes in renal glomeruli [14]. Both *in vitro* and *in vivo* studies indicate that NF-κB and mitogen-activated protein kinase (MAPK) signaling pathways are activated during the progression of renal injury caused by metabolic stresses, subsequently increasing the expression of pro-inflammatory cytokines, such as interleukin (IL)-1β, IL-18, tumor necrosis factor (TNF)-α and transforming growth factor-β1 (TGF-β1). In addition, inflammatory chemokine, such as monocyte chemotactic protein-1 (MCP-1), could also be induced by metabolic stress [15–17]. Moreover, oxidative stress contributes to the pathogenesis of metabolic stress-triggered chronic kidney disease [18]. Nrf-2 controls cellular defense mechanisms against oxidative stress through turning on transcription of antioxidant genes, such as superoxide dismutase 1 (SOD1), NAD(P)H dehydrogenase (quinone 1) (NQO-1) and heme oxygenase-1 (HO-1) [19]. Nrf-2 has been reported to protect against high fat diet- or Fru-induced kidney disease [20]. Therefore, finding effective treatment to reduce the severity of inflammatory and oxidative damage has potential to attenuate Fru-induced kidney injury.

**Figure 1:**
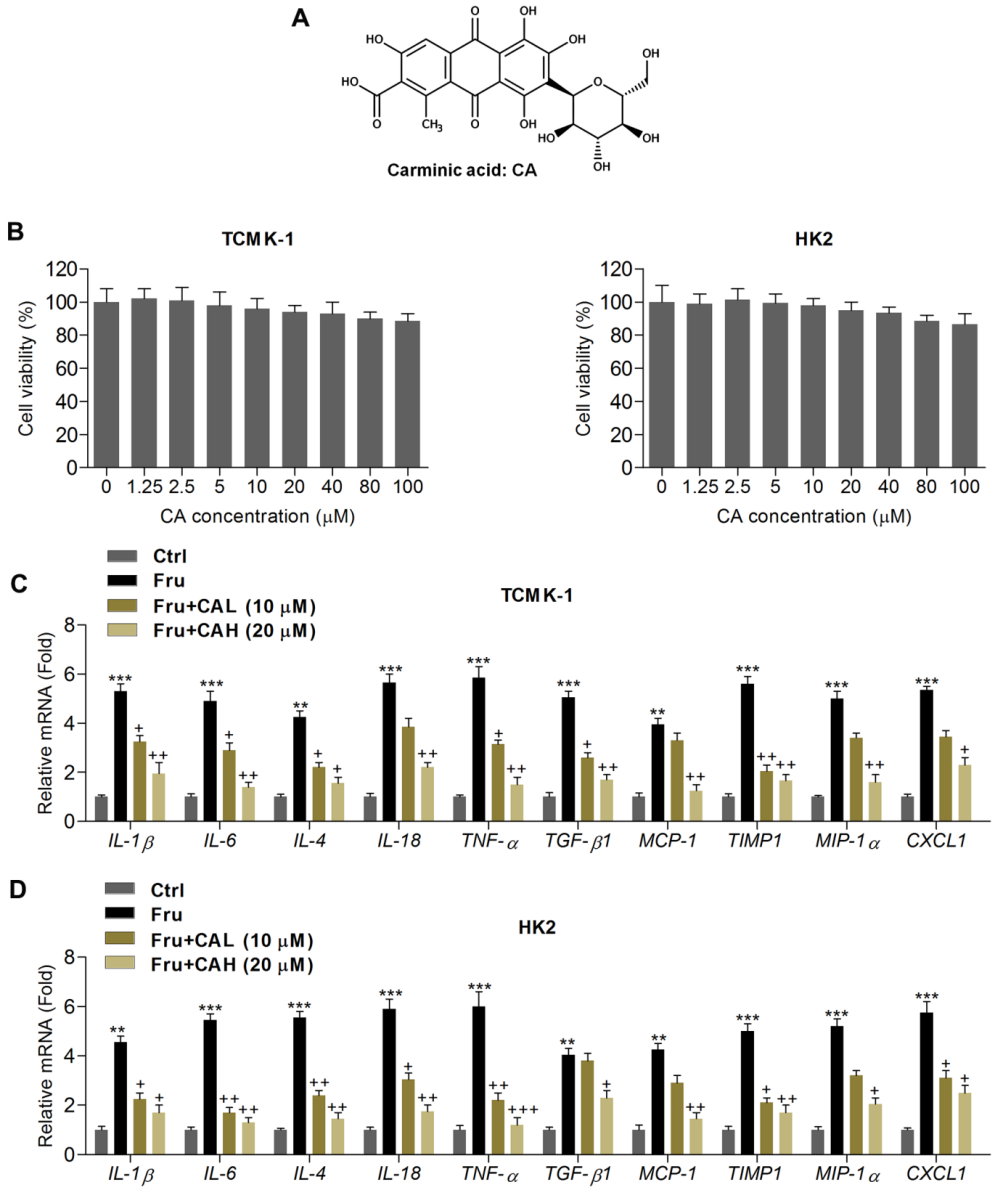
**Carminic acid reduces inflammatory response in Fru-incubated cells.** (**A**) Chemical structure of Carminic acid (CA). (**B**) The mouse tubular epithelial cell line TCMK-1 and human kidney cell line of HK2 were incubated with CA (0, 1.25, 2.5, 5, 10, 20, 40, 80 and 100 μM) for 24 h. Then, all cells were collected for cell viability measurement using MTT analysis. (**C**, **D**) TCMK-1 and HK2 cells were exposed to Fru (5 mM) for 24 h with or without CA (10 and 20 μM). Then, all cells were harvested for the calculation of inflammatory factors using RT-qPCR analysis. The results are expressed as the means ± SEM. n = 4 in each group. ^**^P< 0.01 and ^***^P< 0.001 compared with the Ctrl group; ^+^*P*< 0.05 and ^++^*P*< 0.01 compared with the Fru group.

Carminic acid (CA, 7-β-D-glucopyranosyl-9,10-dihydro-3,5,6,8-tetrahydroxy-1-methyl-9,10-dioxo-2-anthracenecarboxylic acid; Figure 1A), a glucosylated anthraquinone found in scale insects like *Dactylopius coccus*, has been used as a red colorant in different applications since ancient times. CA has a variety of multiple biological activities [21, 22]. CA could inhibit ascites tumors, because its structure is very similar to shikonin and anthracyclines, two anti-cancer drugs. However, CA is not as toxic as shikonin and anthracyclines [23, 24]. Additionally, CA could protect erythrocytes and DNA against radical-elicited oxidation [25]. Furthermore, CA exhibits free radical scavenging activity, herein providing a food additive [26]. According to these potential effects of CA, we supposed that CA might be effective for the treatment of renal dysfunction and injury caused by Fru.

In this study, we for the first time evaluated the cytoprotective effects of CA against Fru-induced injury in mouse tubular epithelial cells and human tubule epithelial cells. The *in vitro* analysis demonstrated that CA treatments markedly reduced the inflammation and ROS production caused by Fru, which was mainly dependent on Nrf-2 signaling. Our animal study confirmed the protective effects of CA against metabolic disorder, renal injury and dysfunction mainly through suppressing inflammation and oxidative stress in Fru-fed mice. Thus, our study identified that CA supplementation may be a potential treatment option for Fru-induced chronic kidney disease.

## RESULTS

### Carminic acid reduces inflammatory response in Fru-incubated cells

In order to calculate the potential of CA on Fru-induced renal injury, the *in vitro* analysis was performed at first. As shown in Figure 1B, MTT analysis showed that CA at the concentrations ranging from 1.25 to 100 μM was non-cytotoxic to TCMK-1 and HK2 cells. Then, 10 and 20 μM of CA were used for the following analysis. Inflammation plays a critical role in inducing renal injury caused by chronic Fru intake [27], and thus we calculated the mRNA expression levels of inflammatory factors by RT-qPCR. As expected, Fru-incubated TCMK-1 and HK2 cells exhibited higher expression of inflammatory cytokines or chemokine, including IL-1β, IL-6, IL-4, IL-18, TNF-α, TGF-β1, MCP-1, tissue inhibitor of metalloproteinase 1 (TIMP-1), macrophage inflammatory protein-1α (MIP-1α) and chemokine (C-X-C motif) ligand 1(CXCL1). However, CA treatment markedly reduced the expression of these factors, demonstrating the alleviated inflammatory response (Figure 1C, 1D). To further explore the potential anti-inflammatory effects of CA, LPS, an inducer for inflammation, was then subjected to TCMK-1 and HK2 cells. As shown in Supplementary Figure 1A, 1B, the mRNA expression levels of IL-1β, IL-6, IL-18 and TNF-α were significantly up-regulated following LPS stimulation. As expected, these results induced by LPS were effectively reversed by CA co-incubation. The findings above confirmed that CA displayed anti-inflammatory abilities.

### Carminic acid blocks the NF-κB and JNK activation in Fru-treated cells

**Figure 2:**
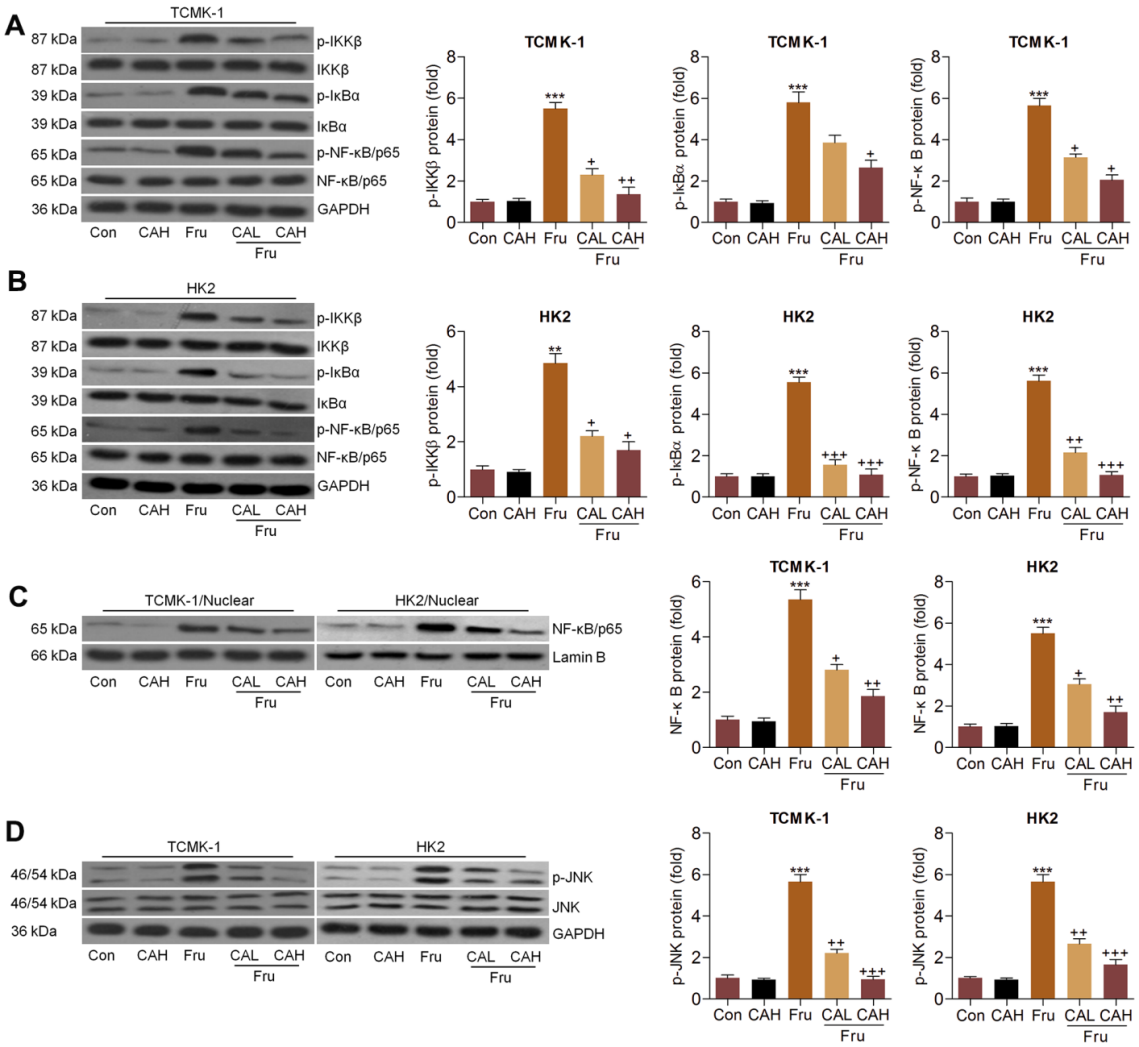
**Carminic acid blocks the NF-κB and JNK activation in Fru-treated cells.** (**A**–**D**) TCMK-1 and HK2 cells were exposed to Fru (5 mM) for 24 h with or without CA (10 and 20 μM). Then, all cells were collected for the subsequent studies. (**A**, **B**) Western blotting analysis for p-IKKβ, p-IκBα and p-NF-κB in TCMK-1 and HK2 cells. (**C**) The protein expression levels of NF-κB were measured in nuclear of cells by western blot analysis. (**D**) Western blot analysis was used to determine p-JNK protein expression levels in cells. The results are expressed as the means ± SEM. n = 4 in each group. ^***^P< 0.001 compared with the Ctrl group; ^+^*P*< 0.05, ^++^*P*< 0.01 and ^+++^*P*< 0.001 compared with the Fru group.

The activation of NF-κB and c-Jun N-terminal kinase (JNK) signaling pathways are critical in inducing inflammation [15, 28], and were then explored. Western blot analysis indicated that Fru significantly activated NF-κB signaling compared to the Ctrl group, as proved by the considerable up-regulation of phosphorylated IκB kinase complex β (p-IKKβ), phosphorylated inhibitors of κB-α (p-IκBα) and p-NF-κB in TCMK-1 and HK2 cells. But CA co-treatment significantly reduced the phosphorylation of IKKβ, IκBα and NF-κB (Figure 2A, 2B). Consistently, NF-κB nuclear translocation stimulated by Fru was also hindered in cells co-treated with CA (Figure 2C), confirming the role of CA in suppressing NF-κB signaling. Moreover, we found that Fru-induced JNK phosphorylation was greatly reduced by CA in renal cells (Figure 2D). Collectively, the data here demonstrated that CA had anti-inflammatory effects in Fru-treated cells through blocking NF-κB and JNK activation.

### Carminic acid suppresses oxidative stress in Fru-cultured cells

**Figure 3:**
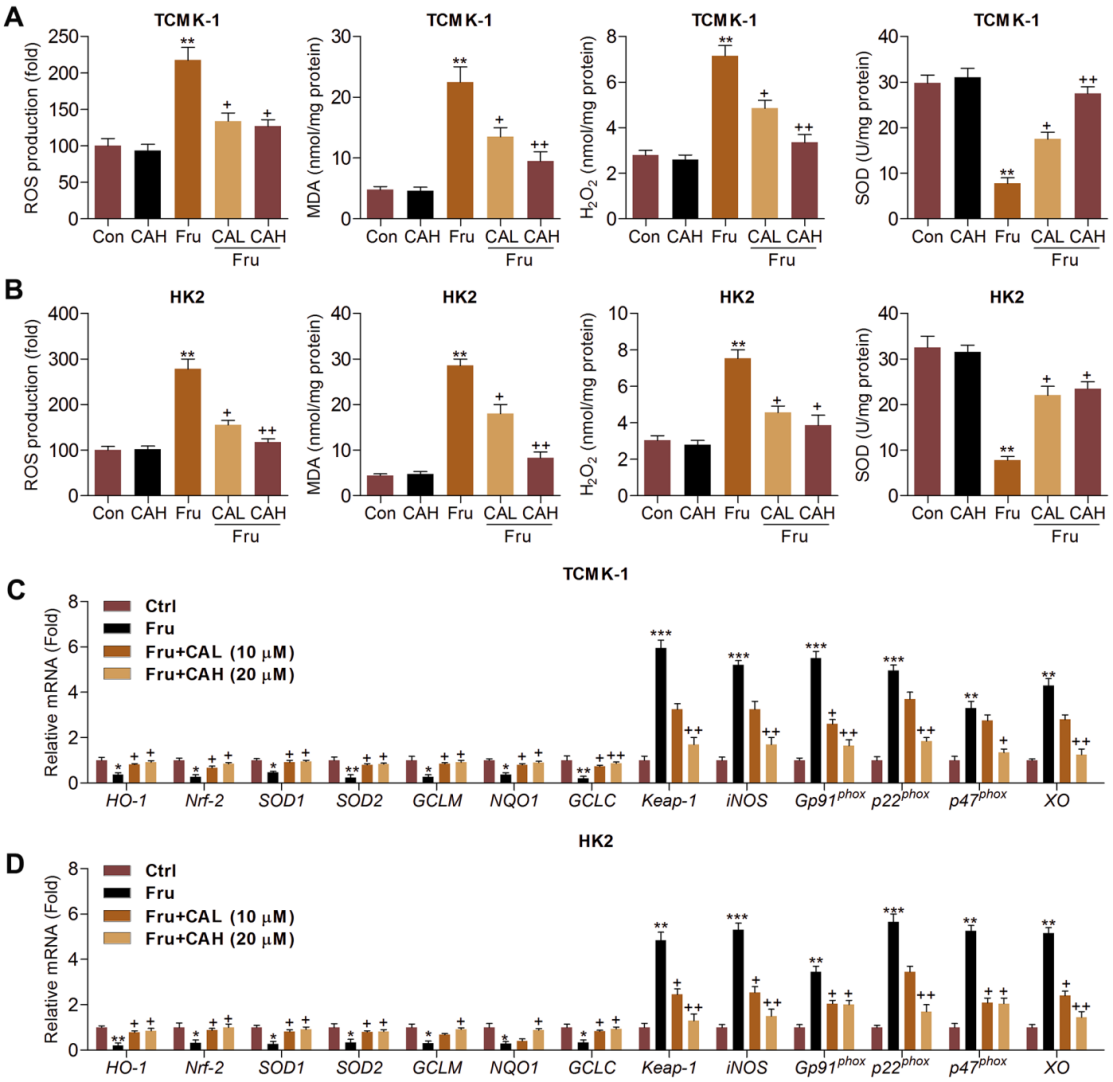
**Carminic acid suppresses oxidative stress in Fru-cultured cells.** TCMK-1 and HK2 cells were exposed to Fru (5 mM) for 24 h with or without CA (10 and 20 μM). Then, all cells were collected for the subsequent studies. Intracellular ROS production, MDA levels, H_2_O_2_ levels and SOD activity were measured in (**A**) TCMK-1 and (**B**) HK2 cells. (**C**, **D**) RT-qPCR analysis was used to measure oxidative stress-associated factors in the cells. The results are expressed as the means ± SEM. n = 4 in each group. ^**^P< 0.01 compared with the Ctrl group; ^+^*P*< 0.05 and ^++^*P*< 0.01 compared with the Fru group.

Oxidative stress contributes to the progression of Fru-triggered renal injury [29], and CA has anti-oxidant biological activity [25, 26]. In this regard, we attempted to investigate if CA could reduce ROS production to ameliorate the development of kidney damage. As illustrated in Figure 3A, 3B, we found that intracellular ROS production, malondialdehyde (MDA) levels and H_2_O_2_ contents were markedly enhanced by Fru, whereas being down-regulated by CA treatment. On the contrary, SOD activity repressed by Fru was greatly rescued due to CA addition. Then, RT-qPCR analysis further showed that Fru significantly reduced the expression of anti-oxidants, including HO-1, Nrf-2, SOD1, SOD2, glutamate-cysteine ligase modifier subunit (GCLM), glutamate-cysteine ligase catalytic subunit (GCLC) and NQO1, while enhanced the expression of Kelch-like ECH-associated protein 1 (Keap-1), inducible nitric oxide synthase (iNOS), Gp91^phox^, p22^phox^, p47^phox^ and xanthine oxidase (XO). However, these effects were effectively reversed by the treatment of CA (Figure 3C, 3D). Together, these findings demonstrated that CA had anti-oxidative effects, which might be involved in the alleviation of renal injury induced by Fru.

### Carminic acid improves Nrf-2 activation in Fru-stimulated cells *in vitro*

**Figure 4:**
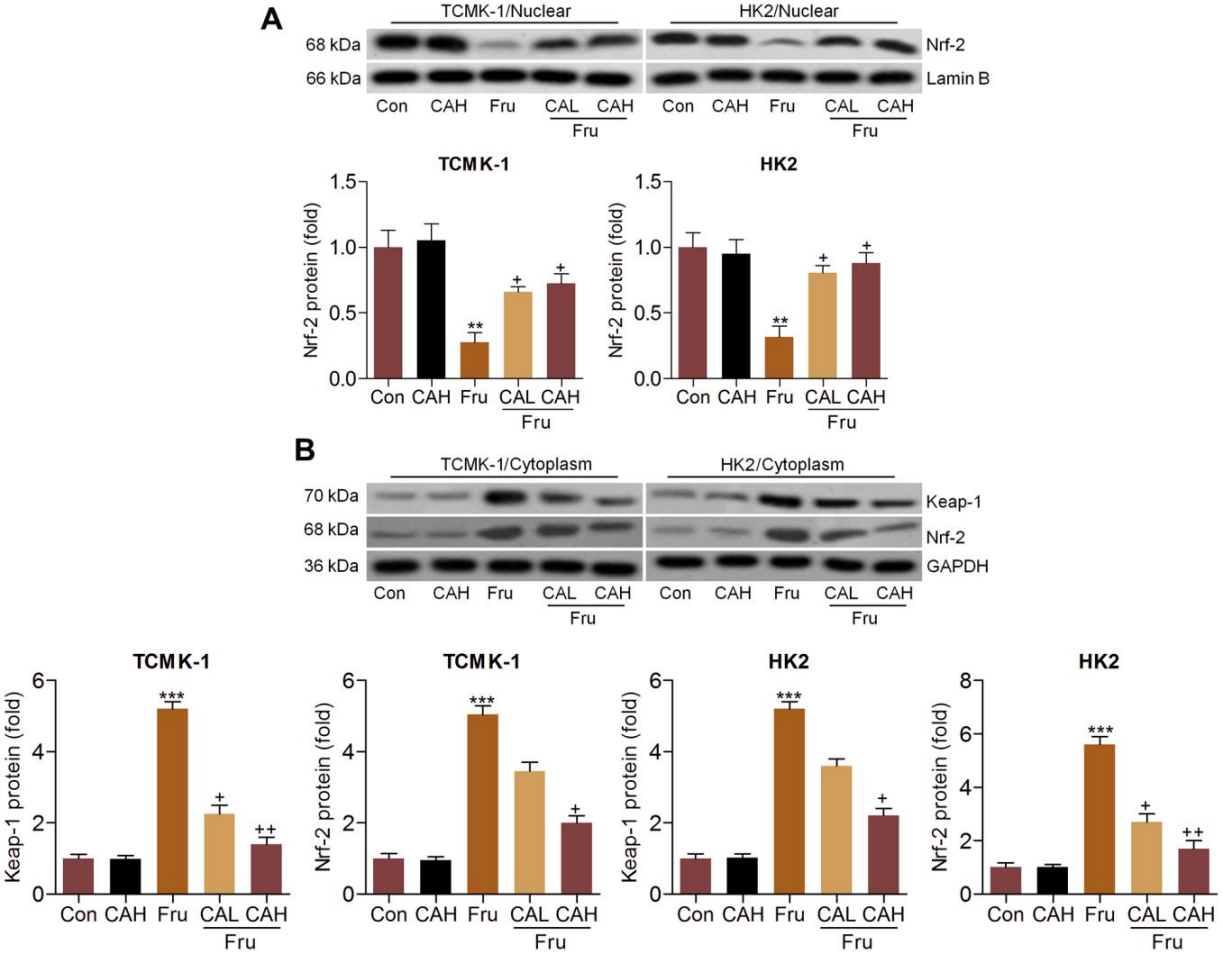
**Carminic acid improves Nrf-2 activation in Fru-stimulated cells *in vitro*.** Western blot analysis was used to determine (**A**) nuclear Nrf-2 and (**B**) cytoplastic Nrf-2 and Keap-1 expression levels in the Fru (5 mM)-incubated TCMK-1 and HK2 cells with or without CA (10 and 20 μM) for 24 h. The results are expressed as the means ± SEM. n = 4 in each group. ^**^P< 0.01 and ^***^P< 0.001 compared with the Ctrl group; ^+^*P*< 0.05 and ^++^*P*< 0.01 compared with the Fru group.

Considering the pivotal role of Nrf-2 in regulating ROS production, we then explored the change of Nrf-2 activation. As shown in Figure 4A, nuclear Nrf-2 was clearly reduced in Fru-treated TCMK-1 and HK2 cells, which were restored by the co-culture of CA. In contrast, the expression levels of Keap-1 and Nrf-2 in cytoplasm were significantly increased following Fru stimulation. But these findings were abolished by CA (Figure 4B). Data in this part suggested that CA could activate Nrf-2 signaling to restrain oxidative stress.

### Carminic acid alleviates renal inflammation and ROS production via Nrf-2 signaling

**Figure 5:**
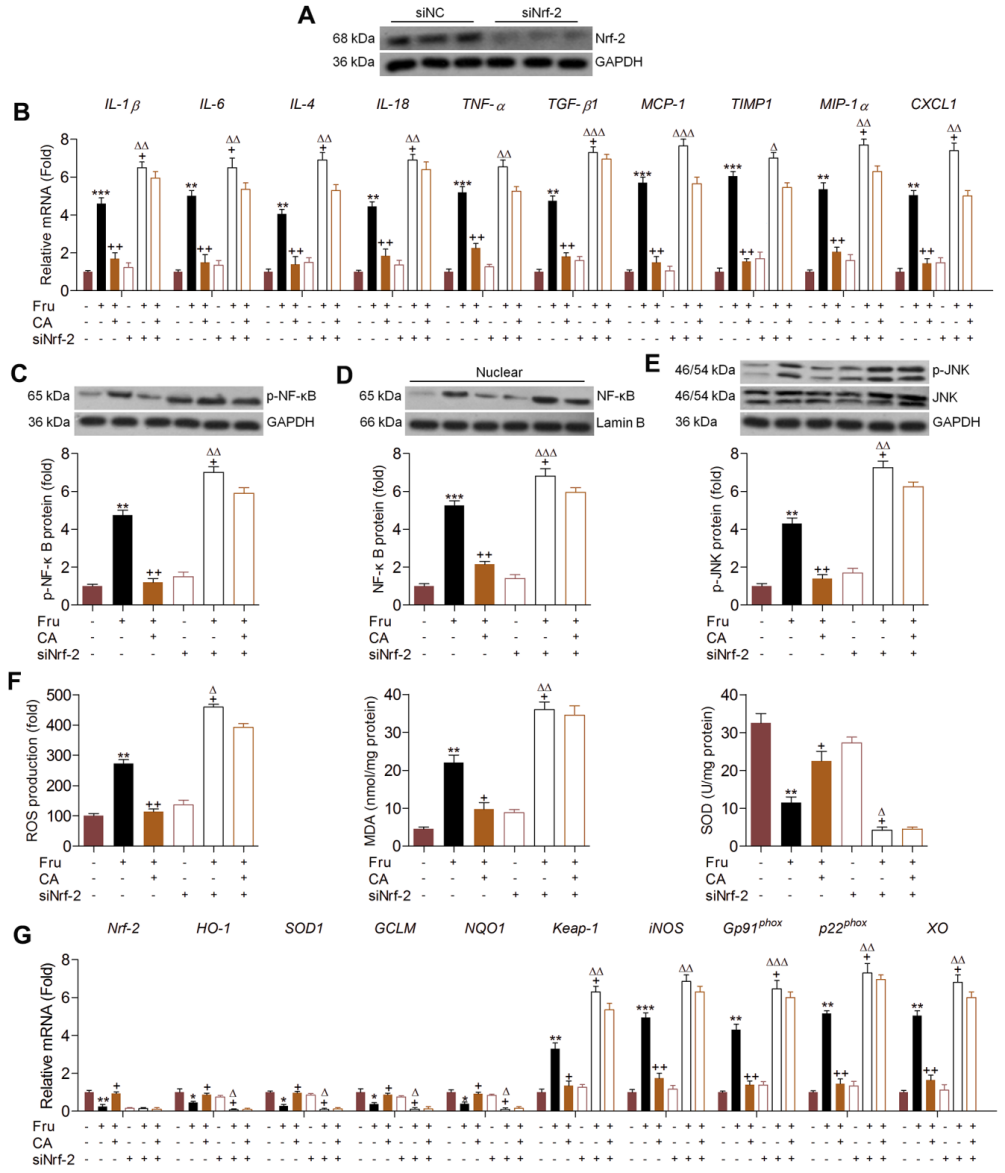
**Carminic acid alleviates renal inflammation and ROS production via Nrf-2 signaling.** (**A**) TCMK-1 cells were transfected with Nrf-2 siRNA for 24 h. Then, the transfection efficiency was measured using western blot analysis. (**B**–**G**) TCMK-1 cells were transfected with siNrf-2 for 24 h, followed by Fru (5 mM) treatment in the absence or presence of CA (20 μM) for another 24 h. Then, all cells were collected for the following analysis. (**B**) The mRNA expression levels of inflammatory factors were measured by RT-qPCR analysis. Western blot analysis of (**C**) cellular p-NF-κB, (**D**) nuclear NF-κB and (**E**) cellular p-JNK. (**F**) ROS production, MDA levels and SOD activities in cells were measured. (**G**) RT-qPCR analysis of genes related to oxidative stress. The results are expressed as the means ± SEM. n = 4 in each group. ^*^P< 0.05, ^**^P< 0.01 and ^***^P< 0.001 compared with the Ctrl group; ^+^*P*< 0.05 and ^++^*P*< 0.01 compared with the Fru group; ^Δ^*P*< 0.05, ^ΔΔ^*P*< 0.01 and ^ΔΔΔ^*P*< 0.001 compared with the siNrf-2 group.

In addition to oxidative stress, Nrf-2 was also reported to modulate inflammation [30]. Therefore, we attempted to explore if CA-regulated renal injury was dependent on Nrf-2 activation. Therefore, Nrf-2 was knocked down by transfection with siNrf-2 (Figure 5A). Then, the effects of CA on inflammation and oxidative stress were investigated in Fru and/or CA-treated TCMK-1 cells with or without Nrf-2 knockdown. As shown in Figure 5B, surprisingly, we found that Fru-induced expression of pro-inflammatory factors was further promoted by siNrf-2. CA-reduced mRNA expression of inflammatory cytokines and chemokine was almost abrogated in Fru-incubated cells with Nrf-2 knockdown. Similarly, Nrf-2 knockdown markedly promoted p-NF-κB expression in Fru-treated cells, accompanied by the evident increase of NF-κB nuclear transition. On the other, siNrf-2 significantly abolished CA-inhibited activation of NF-κB signaling in Fru-treated cells (Figure 5C, 5D). Similarly, Fru-stimulated p-JNK was further accelerated by Nrf-2 knockdown. In Fru-cultured cells, CA-suppressed JNK activation was markedly diminished when Nrf-2 was knocked down (Figure 5E). As expected, Nrf-2 silence markedly enhanced ROS generation in Fru-exposed cells, along with the greatly increased MDA levels. Of note, CA-reduced ROS and MDA in Fru-incubated cells were clearly eliminated by Nrf-2 silence. Opposite results were detected in the change of SOD as shown in Figure 5F. Then, RT-qPCR analysis confirmed that the anti-oxidants including HO-1, SOD1, GCLM and NQO-1 suppressed by Fru were further down-regulated when Nrf-2 was inhibited, while Keap-1, iNOS, p22^phox^ and XO were further accelerated. In accordance with expectation, CA-alleviated oxidative stress was clearly abolished by siNrf-2 in Fru-exposed cells (Figure 5G). Taken together, all findings in this part suggested that CA-attenuated inflammation and oxidative stress induced by Fru were mainly dependent on Nrf-2 signaling.

### Carminic acid ameliorates renal dysfunction in Fru-fed mice

The *in vitro* analysis demonstrated that CA exerted anti-inflammatory and anti-oxidant activities in Fru-treated cells, demonstrating its potential in alleviating renal injury caused by chronic Fru intake. To further investigate the effects of CA on Fru-triggered kidney damage and the underlying molecular mechanisms, the *in vivo* analysis was then performed. At first, the safety of CA was explored. As displayed in Supplementary Figure 2A, there was no significant difference observed in the histological alterations of heart, liver, lung and spleen between the Con and CAH groups. Moreover, the contents of serum ALT, AST and ALP that reflect hepatic functions were not markedly changed in CAH mice compared with the Con group of mice (Supplementary Figure 2B). Therefore, CA at the concentrations used in our study showed little toxicity.

**Figure 6:**
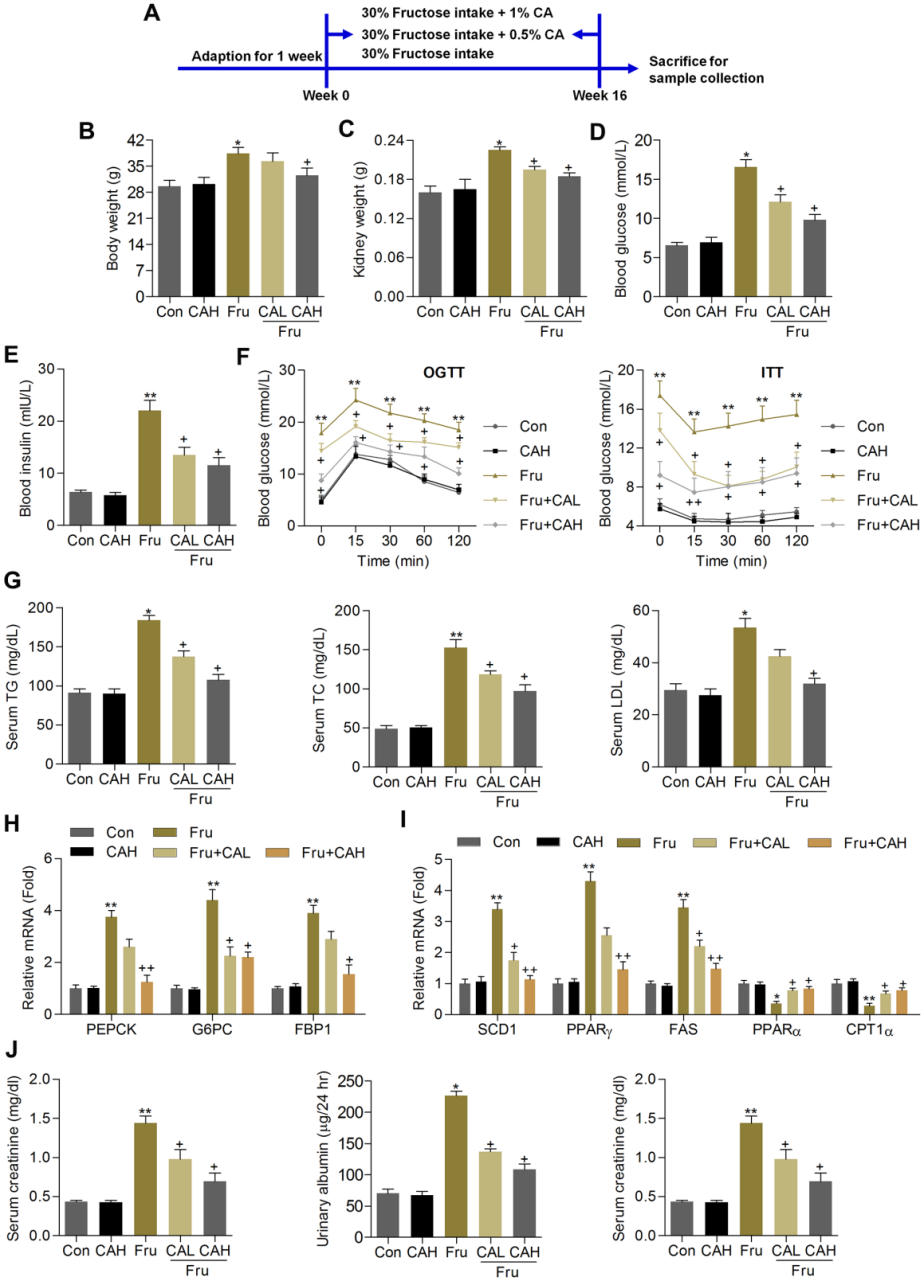
**Carminic acid ameliorates renal dysfunction in Fru-fed mice.** (**A**) Animal experimental procedure was shown. (**B**) Body weight of mice. (**C**) Kidney weight of mice was measured. n = 8 in each group. (**D**) Blood glucose levels were assessed. (**E**) Serum insulin levels were measured. (**F**) OGTT and ITT analysis were performed. n = 8 in each group. (**G**) Serum TG, TC and LDL contents were determined. n = 8 in each group. (**H**) RT-qPCR analysis for gene expression of PEPCK, G6PC and FPB1 in kidney samples of mice. n = 4 in each group. (**I**) RT-qPCR results for genes regulating fatty acid synthesis (SCD1, PPARγ and FAS) and β-oxidation (PPARα and CPT1α) in renal tissues. n = 4 in each group. (**J**) Serum creatinine contents, urinary albumin and BUN levels were determined. n = 8 in each group. The results are expressed as the means ± SEM. ^*^P< 0.05and ^**^P< 0.01 compared with the Ctrl group; ^+^*P*< 0.05 compared with the Fru group.

Animal experiment procedure was shown in Figure 6A. We found that compared with the Con group, chronic Fru intake significantly increased the body weight, kidney weight and blood glucose levels, which were however markedly attenuated by CA supplementation (Figure 6B–6D). Subsequently, ELISA analysis demonstrated that mice with Fru treatment had higher serum insulin levels than that of the Con group, while being reduced by CA administration (Figure 6E). As expected, mice developed more severe glucose intolerance and insulin resistance upon Fru challenge than the Con group of mice, which were markedly alleviated by CA treatment, as revealed by the OGTT and ITT analysis (Figure 6F). In addition, serum TG, TC and LDL contents were highly induced by Fru, and CA treatments significantly reversed these results (Figure 6G). We then found that CA supplementation significantly decreased the mRNA expression levels of the major gluconeogenesis-related factors including phosphoenolpyruvate carboxykinase 1 (PEPCK), glucose-6-phosphatase (G6PC) and fructose bisphosphatase 1 (FBP1) in kidney samples of mice challenged with Fru (Figure 6H). Additionally, RT-qPCR results demonstrated that excessive Fru intake significantly increased the mRNA expression levels of genes associated with lipid synthesis including stearoyl-CoA desaturase 1 (SCD1), peroxisome proliferation-activated receptor γ (PPARγ) and fatty acid synthase (FAS) in kidney samples, and decreased the expression of genes controlling fatty acid β-oxidation including PPARα and carnitine palmitoyltransferase-1α (CPT1α) (Figure 6I). These data indicated that CA could alleviate Fru-triggered metabolic disorder in mice by improving glucose and lipid metabolism.

Because albuminuria reflects renal dysfunction during metabolic stress-induced kidney injury, including Fru [31], and serum creatinine and BUN are another 2 hallmarks of renal injury [32, 33]. We then found that the contents of serum creatinine, urinary albumin and BUN increased by Fru were effectively reduced in mice co-treated with CA (Figure 6J). Therefore, CA might be effective for ameliorating renal dysfunction caused by metabolic stress.

### Carminic acid attenuates the pathological symptoms in the Fru-induced chronic renal injury

**Figure 7:**
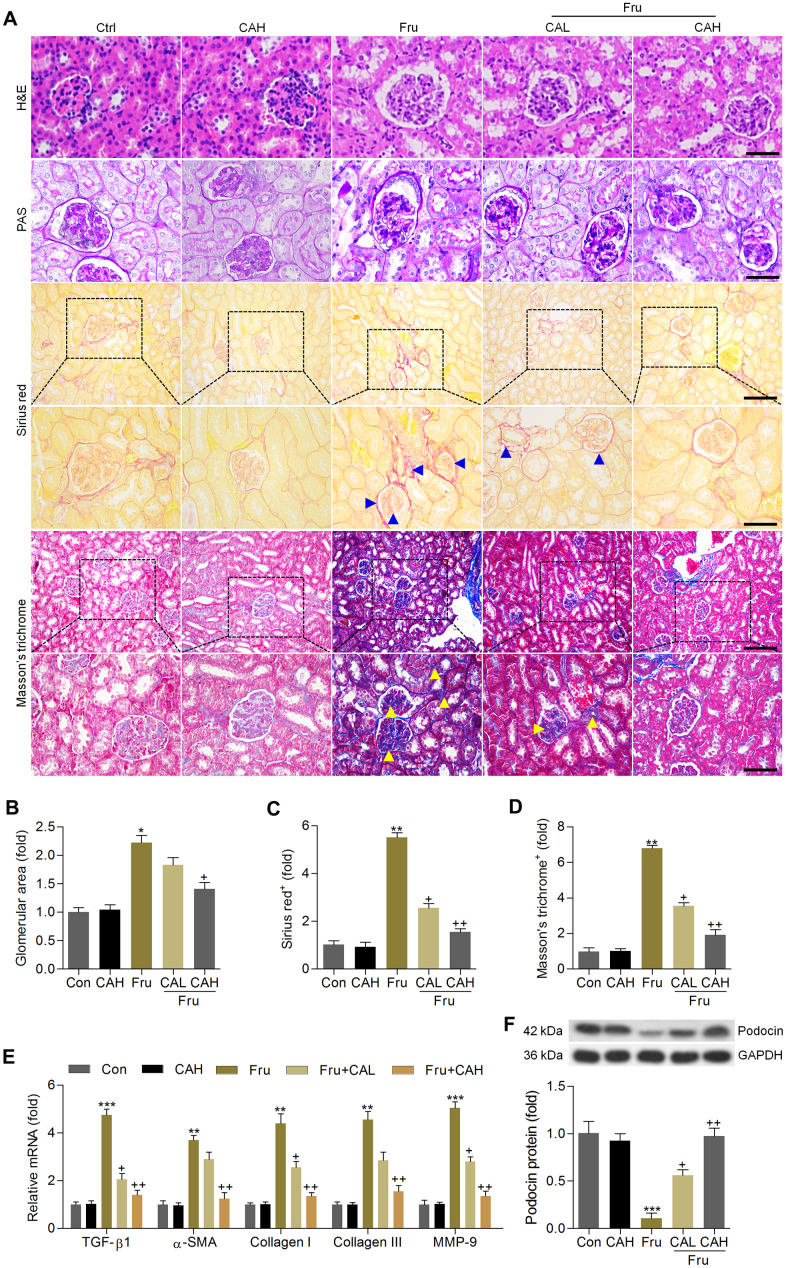
**Carminic acid attenuates the pathological symptoms in the Fru-induced chronic renal injury.** (**A**) H&E (Scale bar = 50 μm), PAS (Scale bar = 50 μm), Sirius red (blue arrow: collagen accumulation; Up panel, Scale bar = 100 μm; Down panel, Scale bar = 50 μm) and Masson’s trichrome (yellow arrow: collagen accumulation; Up panel, Scale bar = 100 μm; Down panel, Scale bar = 50 μm) staining of renal sections for the calculation of histological changes. (**B**) Glomerular area was quantified. Calculation of fibrosis condition following (**C**) Sirius red and (**D**) Masson’s trichrome staining. (**E**) RT-qPCR analysis for fibrotic genes in renal tissues. (**F**) Western blot analysis of podocin in kidney samples. The results are expressed as the means ± SEM. n = 4 in each group.^*^P< 0.05, ^**^P< 0.01 and ^***^P< 0.001 compared with the Ctrl group; ^+^*P*< 0.05 and ^++^*P*< 0.01 compared with the Fru group.

H&E and PAS staining demonstrated that compared with the Con group, Fru-fed mice had notable glomerular hypertrophy, whereas these hypertrophic changes were markedly reduced in CA-treated groups (Figure 7A, 7B). Sirius red and Masson’s trichrome staining showed that Fru led to collagen accumulation mainly in glomeruli and tubulointerstitium (as arrows indicated), and CA improved these histologic changes of the kidneys (Figure 7A, 7C, 7D). Similar anti-fibrotic effects of CA were observed by the RT-qPCR analysis, as evidenced by the significantly reduced mRNA levels of TGF-β1, α-smooth muscle actin (α-SMA), collagen I, collagen III and matrix-metalloproteinase-9 (MMP-9) in renal tissues (Figure 7E). To further confirm the potential anti-fibrotic abilities of CA, the *in vitro* studies were conducted in cells incubated with or without TGF-β. As shown in Supplementary Figure 1C, 1D. RT-qPCR analysis indicated that cells with TGF-β stimulation exhibited higher expression of α-SMA, collagen I, collagen III and MMP-9 than that of the Con group, which were, however, decreased by CA co-incubation, further demonstrating the anti-fibrotic effects of CA that were independent of glycemic control. The decrease of podocin is a starting point for progressive kidney disease [34]. Western blot analysis demonstrated that podocin expression levels were markedly reduced in kidneys of Fru-challenged mice, while being improved in mice supplemented with CA (Figure 7F). Altogether, these findings indicated that CA had protective effects against Fru-induced renal injury.

### Carminic acid inhibits Fru-induced inflammation in kidney of Fru-challenged mice

**Figure 8:**
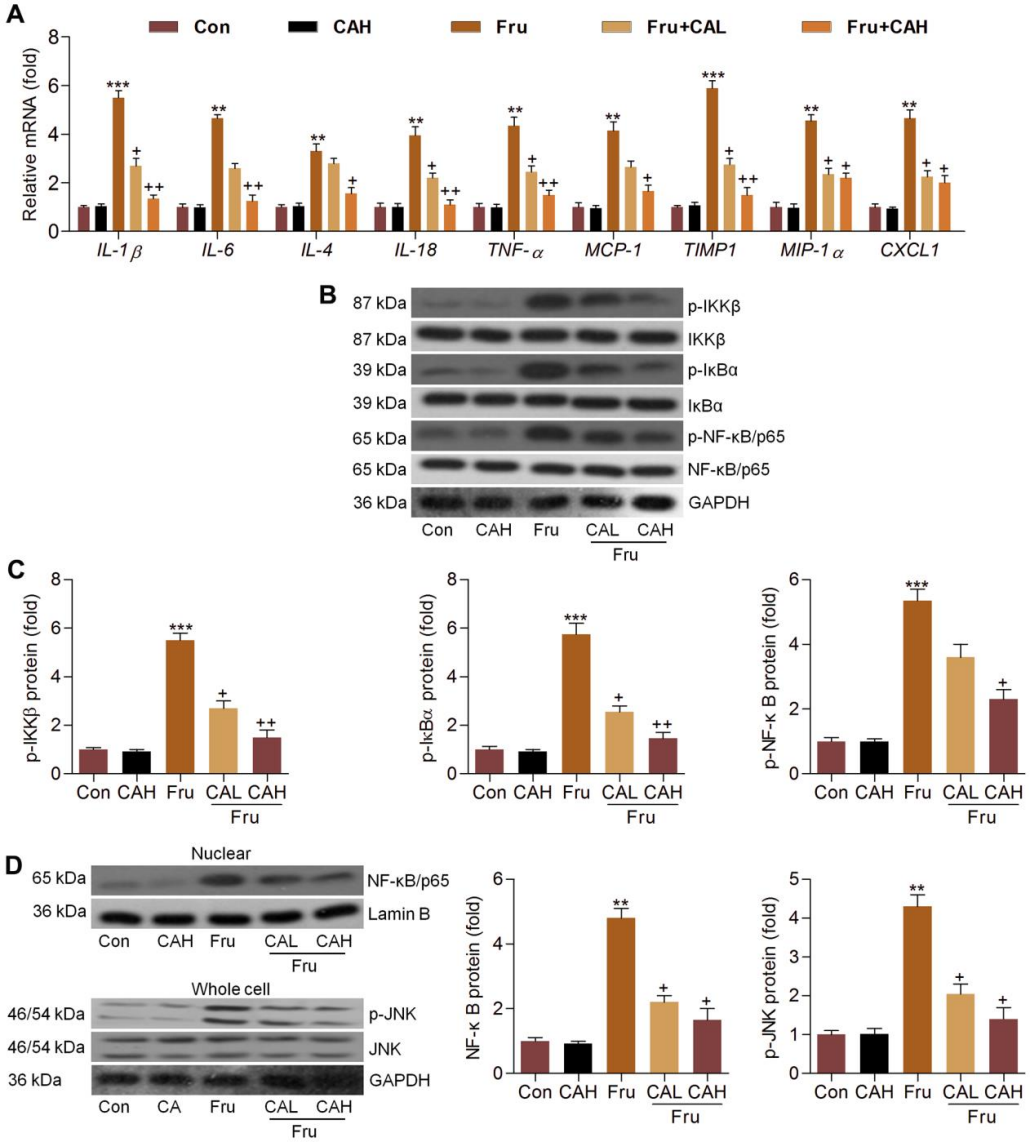
**Carminic acid inhibits Fru-induced inflammation in kidney of Fru-challenged mice.** (**A**) The mRNA expression levels of inflammatory factors in kidney tissues were measured by RT-qPCR analysis. (**B**, **C**) Western blot analysis was used to determine p-IKKβ, p-IκBα and p-NF-κB protein expression levels in kidney. (**D**) Nuclear NF-κB and whole cell p-JNK protein expression levels were assessed by western blot analysis. The results are expressed as the means ± SEM. n = 4 in each group. ^**^P< 0.01 and ^***^P< 0.001 compared with the Ctrl group; ^+^*P*< 0.05 and ^++^*P*< 0.01 compared with the Fru group.

In this section, RT-qPCR analysis suggested that Fru-challenged mice had higher expression levels of inflammatory factors, such as IL-1β, IL-6, IL-4, IL-18, TNF-α, MCP-1, TIMP1, MIP-1α and CXCL1. As detected *in vitro*, CA treatment markedly reduced the mRNA levels of these molecules (Figure 8A). Moreover, Fru-activated IKKβ, IκBα and NF-κB were significantly abolished by CA (Figure 8B, 8C). Also, NF-κB nuclear translocation and JNK activation enhanced by Fru were evidently abrogated by CA supplementation (Figure 8D). These *in vivo* results confirmed the anti-inflammatory effects of CA against Fru-induced renal injury.

### Carminic acid hinds oxidative stress by improving Nrf-2 signaling in kidneys of Fru-treated mice

**Figure 9:**
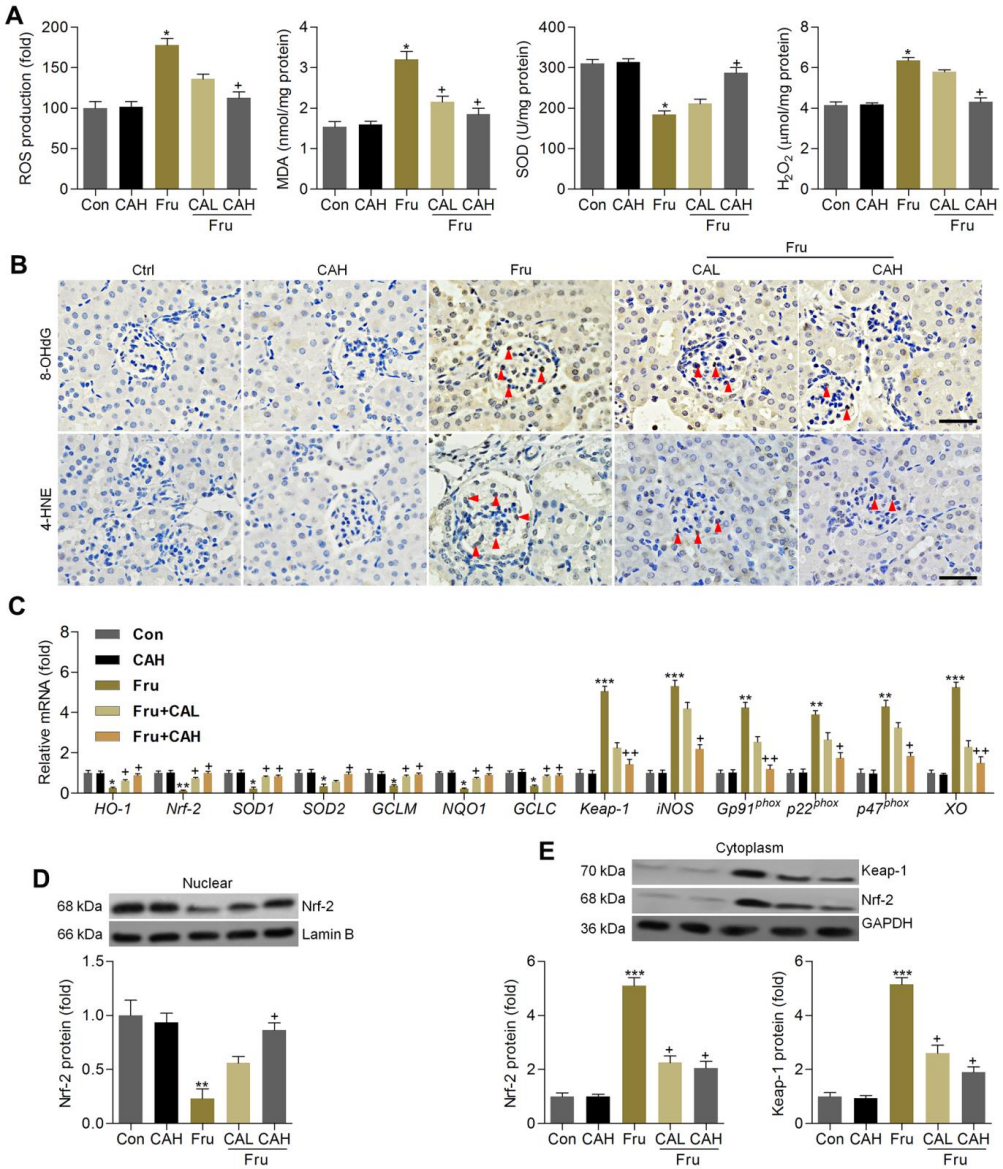
**Carminic acid hinds oxidative stress by improving Nrf-2 signaling in kidney of Fru-treated mice.** (**A**) Assessments of ROS production, MDA levels, SOD activity and H_2_O_2_ levels in kidney samples of mice. n = 8 in each group. (**B**) Immunohistochemistry analysis for 8-OHdG and 4-HNE in renal sections (Scale bar = 50 μm). Red arrows indicated the positive-staining area. n = 4 in each group. (**C**) Oxidative stress-associated genes were measured by RT-qPCR analysis. n = 4 in each group. (**D**) Nuclear Nrf-2, (**E**) cytoplastic Keap-1 and Nrf-2 protein expression levels were evaluated using western blotting assays. n = 4 in each group. The results are expressed as the means ± SEM. ^*^P< 0.05, ^**^P< 0.01 and ^**^P< 0.001 compared with the Ctrl group; ^+^*P*< 0.05 and ^++^*P*< 0.01 compared with the Fru group.

The anti-oxidative role of CA was further explored in kidneys of Fru-challenged mice. As shown in Figure 9A, ROS production, MDA levels and H_2_O_2_ contents in renal tissues were markedly up-regulated in Fru-fed mice, whereas SOD activity was down-regulated. However, these findings were clearly reversed by CA. 8-OHdG and 4-HNE are well-known markers for oxidative stress [35], and thus were investigated. Immunohistochemistry analysis demonstrated that Fru challenge increased the expression of 8-OHdG and 4-HNE in renal sections mainly in the glomerulus areas, which were evidently attenuated by CA treatments (Figure 9B). Consistently, Fru-decreased mRNA levels of HO-1, Nrf-2, SOD1, SOD2, GCLM, NQO-1 and GCLC were greatly rescued by CA, and opposite results were observed in the expression changes of Keap-1, iNOS, Gp91^phox^, p22^phox^, p47^phox^ and XO (Figure 9C). Western blot analysis demonstrated that Nrf-2 nuclear expression levels were highly restored in kidneys of Fru-treated mice (Figure 9D). On the contrary, Keap-1 and Nrf-2 expression levels in cytoplasm of renal samples were highly increased by Fru, while being decreased in mice co-treated with CA (Figure 9E). Therefore, findings above showed that CA-attenuated renal injury caused by Fru might be associated with its anti-oxidative ability.

### Carminic acid improves AMPKα activation in Fru-treated cells and renal samples

**Figure 10:**
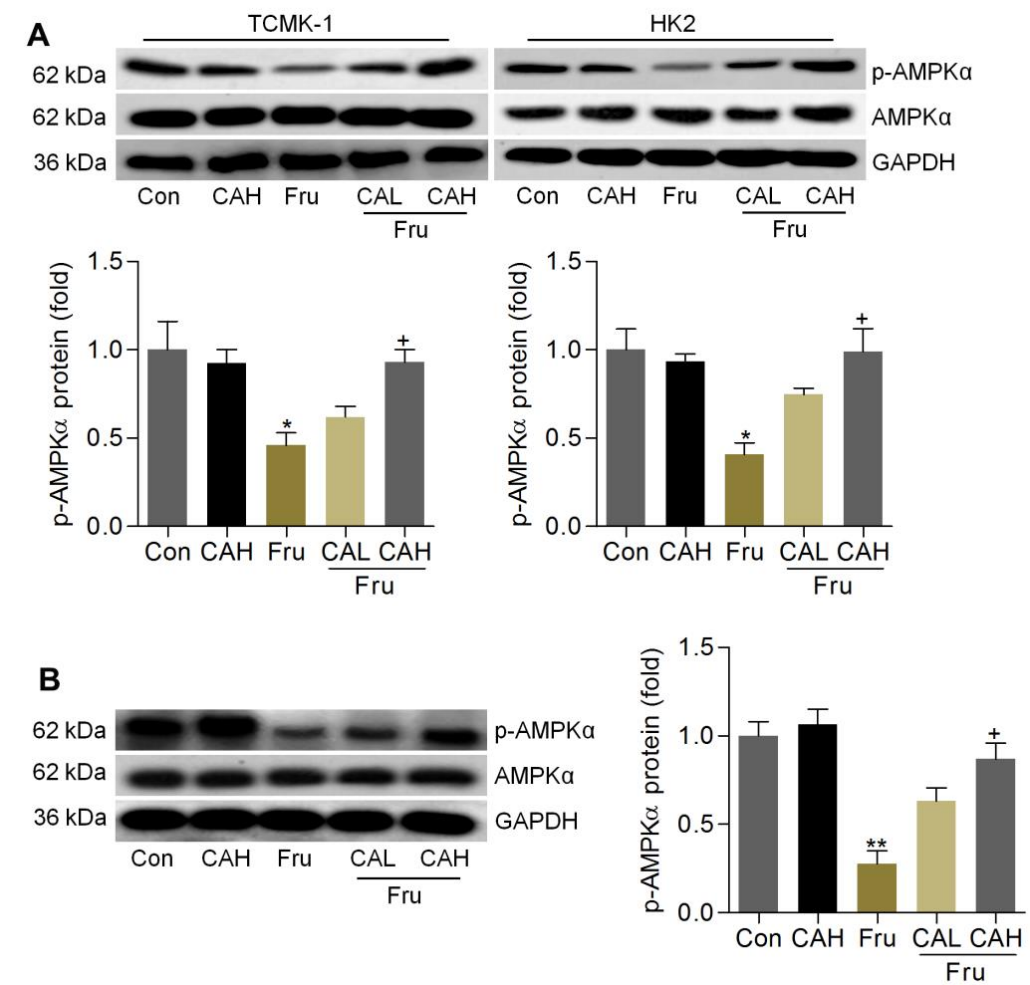
**Carminic acid improves AMPKα activation in Fru-treated cells and renal samples.** (**A**) TCMK-1 and HK2 cells were exposed to Fru (5 mM) for 24 h with or without CA (10 and 20 μM). Then, all cells were collected for western blot analysis of p-AMPKα in cells. n = 4 in each group. (**B**) Western blot results for p-AMPKα in kidney samples of mice from the indicated groups. n = 4 in each group. The results are expressed as the means ± SEM. ^*^P< 0.05 and ^**^P< 0.01 compared with the Ctrl group; ^+^*P*< 0.05 compared with the Fru group.

As Fur-triggered renal injury is a metabolic disorder, and AMP-activated protein kinase α (AMPKα) activation is involved in the event [36]. To explore if the protective effects of CA against Fru-induced kidney injury was associated with AMPKα activation, western blotting analysis was then performed. As shown in Figure 10A, we found that AMPKα activation was markedly blocked in Fru-treated cells, while being mitigated by CA incubation, as evidenced by the up-regulated expression of p-AMPKα. Consistently, mice with Fru challenge exhibited significantly reduced expression of p-AMPKα in renal tissues, and this result was effectively rescued by CA supplementation (Figure 10B). Therefore, results above showed that CA-alleviated renal injury and metabolic disorder were associated with the improvement of AMPKα activation.

## DISCUSSION

Renal injury caused by excessive Fru consumption has become an increasing public health issue [1, 2]. The hallmarks of kidney disease caused by metabolic stresses such as Fru and high fat diet include scarring of the glomeruli, proteinuria, and a progressive reduction in kidney function [31–33]. The main histological characteristics of the disease include glomerular basement membrane thickening, glomerular mesangial expansion, glomerular sclerosis, and progressive general fibrosis [37, 38]. Furthermore, hyperglycemia can drive the progression of Fru-induced chronic kidney disease [16]. Although great advantages have been made to improve the diagnosis and treatment of kidney injury associated with metabolic syndrome, the nephropathy is often progressive without effective therapies [10, 11]. Oxidative stress and its constant companion inflammatory response are common features and major regulators during the progression of Fru-induced renal injury, as well as the associated complications [12, 15, 16, 18]. Therefore, factors or chemical drugs that control these signaling pathways may be attractive options.

**Figure 11:**
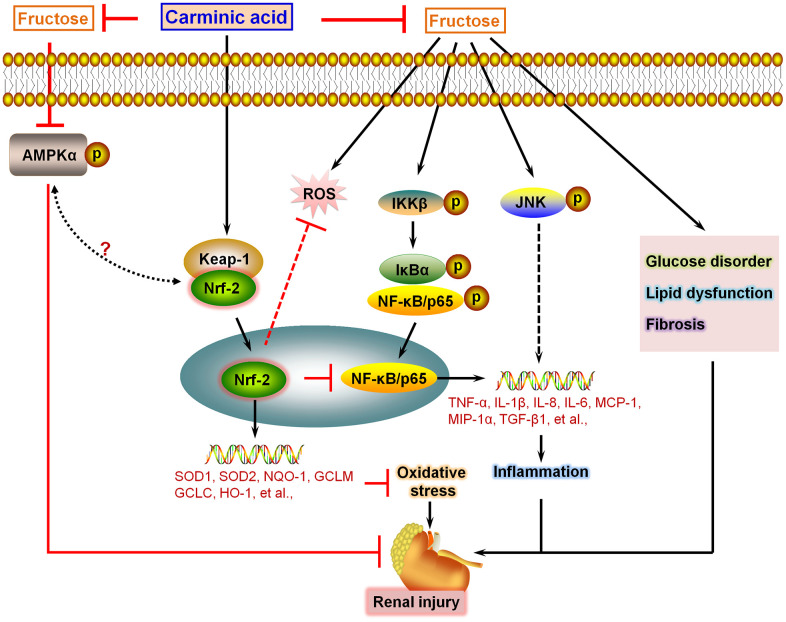
**Schematic diagram of proposed mechanism by which Carminic acid alleviates fructose-induced kidney injury.** On the basis of our findings in this study and our published data in the study of chronic kidney injury, we proposed that Fru could induce glucose disorder, dyslipidemia, AMPKα inactivation and fibrosis in renal tissues of mice. In addition, we showed that Fru treatment led to significant inflammatory response in cells and in kidney tissues of mice through promoting the activation of NF-κB and JNK signaling pathways. Moreover, oxidative stress was also induced by Fru both *in vitro* and *in vivo*. Notably, we found that CA treatment could reverse all these events caused by Fru, alleviating chronic kidney injury consequently. Importantly, we demonstrated that CA-alleviated inflammation and oxidative stress were mainly dependent on Nrf2 activation.

In this study, we for the first time reported that CA could effectively suppress inflammatory response and ROS generation in Fru-incubated cells via activating Nrf-2 signaling, eventually reversing cell injury. In Fru-fed mice for 16 weeks, we further demonstrated that CA treatment attenuated metabolic disorders, as evidenced by the significantly reduced blood glucose levels and serum lipid contents. These events were partly associated with the down-regulation of gluconeogenesis through reducing PEPCK, G6PC and FBP1. Meanwhile, fatty acid synthesis induced by Fru in renal tissues was also alleviated by CA, along with improved fatty acid β-oxidation. Fru-challenged mice showed higher serum creatinine, urinary albumin and BUN, demonstrating the renal dysfunction. Histological results suggested that Fru intake promoted glomerular and fibrotic areas in kidneys of mice, and fibrosis is reported as the most fundamental and prominent feature of nephropathy [39]. Under fibrotic conditions, excessive secretion of extracellular matrix components (ECM), such as collagen I, α-SMA and MMP-9, promotes the pathological process of renal fibrosis, leading to metabolic syndrome-associated renal disease [17, 40]. Notably, we found that CA supplementation could significantly alleviate Fru-induced renal dysfunction and fibrosis by reducing TGF-β1, collagen I, collagen III, α-SMA and MMP-9 expression levels. The anti-fibrotic ability of CA was verified in TGF-β-stimulated cells. Consistent with the *in vitro* results, Fru led to significant inflammation and oxidative stress in renal tissues, which were also repressed by CA administration. Moreover, AMPKα activation was restrained by Fru both *in vitro* and *in vivo*, whereas being improved in response to CA treatment. In the end, H&E staining and biochemical analysis indicated that CA at the concentrations used in our present study had few side effects or toxicities. Collectively, all these findings supported that CA may be a potential therapeutic strategy against Fru-induced kidney injury (Figure 11).

An increasing number of clinical and animal studies have demonstrated that inflammatory response is involved in the pathogenesis of kidney damage induced by Fru [12, 14, 41]. The NF-κB signaling pathway plays a critical role in inducing inflammatory response, contributing to the releases of inflammatory cytokines and chemokine [15, 42, 43]. When inactive, NF-κB dimers are sequestered in the cytoplasm by a family of inhibitors known as IκBs, especially IκBα. IκBα could be activated when it is phosphorylated, leading to its dissociation from the NF-κB cytoplasmic complex. Once being activated, the subunit of NF-κB, known as p65, translocates into the nucleus, contributing to the expression of its down-streaming targets associated with pro-inflammatory response [44, 45]. It has been illustrated that Fru intake can promote NF-κB transcription by increasing phosphorylation of IκB [46]. Moreover, JNK can be activated by numerous factors of the diabetic milieu, such as hyperglycemia, advanced glycation end-products, oxidative stress, and pro-inflammatory factors [47, 48]. Therefore, promotion of inflammatory signaling pathways is an essential factor that contributes to the development of Fru-triggered nephropathy [12, 14]. In our study, we confirmed that Fru led to inflammatory response in cells and in kidneys of mice, which was mainly through the activation of NF-κB signaling, as evidenced by the markedly up-regulated expression of p-IKKβ, p-IκBα and p-NF-κB. At the same time, nuclear NF-κB transition and p-JNK were highly induced by Fru. However, CA treatment could effectively block NF-κB and JNK activation to subsequently repress inflammation, alleviating renal injury eventually. In addition to Fru treatment, our *in vitro* studies showed that the elevated expression levels of pro-inflammatory cytokines were markedly decreased by CA in LPS-stimulated cells, confirming the anti-inflammatory effects of CA.

Oxidative stress contributes to the pathogenesis of chronic kidney disease caused by Fru. Elevated ROS production in kidney is accompanied with a disturbed endogenous antioxidant defense system. Previous pharmacological analysis has focused on reducing ROS or boosting cellular antioxidants [18, 20, 29]. Nrf-2/Keap-1 system controls the antioxidant redox signaling to repress the initiation of diabetes mellitus [49]. Increasing studies have indicated that Nrf-2 is a cytoprotective transcription factor, which has an essential role in maintaining the basal activity by coordinating the generation of target genes, including antioxidant enzymes (SOD1, SOD2, HO-1, et al), the critical enzymes responsible for glutathione synthesis (GCLC and GCLM), and the major detoxifying enzyme NQO-1 [19, 20, 50]. Others have indicated the beneficial effects of Nrf-2 induction in suppressing renal injury induced by Fru [20]. In our study, we confirmed that Fru treatment led to oxidative stress and excessive ROS *in vitro* and *in vivo*, contributing to the progression of renal injury. Notably, CA, as reported previously [25, 26], exhibited anti-oxidative effects to ameliorate kidney injury through improving Nrf-2 activation, as evidenced by its higher nuclear translocation. Moreover, Nrf-2 is involved in the regulation of NF-κB signaling, and thus is linked to the pro-inflammatory response under different conditions, including metabolic diseases [51–53]. Based on the *in vitro* results, we found that Fru-induced inflammation and oxidative stress were further accelerated by Nrf-2 knockdown. Of note, CA-alleviated pro-inflammatory response and ROS generation were almost abrogated in Fru-treated cells transfected with siNrf-2. Herein, we concluded that Nrf-2 was necessary for CA to attenuate inflammatory injury and oxidative damage induced by Fru. AMPKα signaling is a classic key molecular pathway revealing the coordinated control of nutrient balance [40, 54, 55]. Furthermore, AMPKα, as a kinase that works up-streaming of Nrf2, has attracted attention for its relationship with redox homeostasis and energy metabolism [56]. Activation of AMPK has been shown to reduce fat accumulation and increase glucose tolerance, insulin sensitivity, mitochondrial biogenesis, and physical endurance [57–59]. Here, we consistently found that Fru stimulation restrained AMPKα activation both in cells and in kidneys, which were found to be rescued by CA. Therefore, we supposed that CA-alleviated renal injury caused by Fru might be associated with the improvement of AMPKα activation. Recently, a potential crosstalk between AMPKα and Nrf2 signaling pathways has been reported under multiple different conditions, including metabolic stress-induced chronic kidney injury [54, 60, 61]. Thus, we could not exclude that CA-improved Nrf2 signaling might be related to AMPKα molecular pathway. As for this, further investigation in future is necessary for our group.

In conclusion, we for the first time demonstrated that CA, functioning as a potential Nrf-2 activator, significantly suppressed oxidative stress and inflammation regulated by NF-κB and JNK in Fru-challenged samples, and these events were largely dependent on Nrf2 signaling. Moreover, Fru-triggered glucose disorder, lipid accumulation and fibrosis were also mitigated in CA-treated mice, accompanied with AMPKα activation. All these solid evidence indicated that CA supplementation could alleviate Fru-induced kidney injury with few side effects (Figure 11). Collectively, CA may be useful and effective as a therapeutic strategy for the treatment of chronic renal injury caused by excessive Fru consumption. Nevertheless, more studies are still required to further elucidate the involved molecular mechanisms and the safety of CA for clinical application.

## MATERIALS AND METHODS

### Cells and culture

HK2, an immortalized proximal tubule epithelial cell line from the adult human kidney, was purchased from the Cell Bank of the Chinese Academy of Science (Shanghai, P.R. China). The mouse tubular epithelial cell line TCMK-1 (#CCL-139TM) was purchased from American Type Culture Collection (ATCC, Manassas, VA, USA). All cells were cultured in RPMI1640 medium (Wisent Corporation, Nanjing, China) containing 10% fetal bovine serum (FBS, Gibco Corporation, USA) and 1 × 10^5^ U/L streptomycin sulfate (Gibco) in a constant environment of 37° C with 5% CO_2_. The cells were then incubated with Fru in the absence or presence of CA (HPLC ≥ 95%, Sensient Technologies, Guangzhou, China). The negative control (NC) and Nrf-2-specific siRNAs (siNrf-2) were synthesized and purchased from Shanghai Generay Biotech (Shanghai, China) and transfected into cells using Lipofectamine® 3000 (Invitrogen, USA) according to the manufacturer’s protocols. In order to induce inflammation and fibrosis *in vitro*, lipopolysaccharide (LPS; #L8880, Solarbio, Beijing, China) and Recombinant Human TGF-β (#P00121, Solarbio) or Mouse TGF-β (#P00199, Solarbio) were exposed to cells.

### Cell viability analysis

The 3-(4-5-dimethylthiazol-2-yl-2,5-diphenyltetrazolium bromide (MTT) analysis (Beyotime, Nanjing, China) was used to assess the survival of the cells after CA incubation. In brief, the cells were planted in 96-well plates (1 × 10^4^ cells/well). After treatment with CA, the cells were exposed to MTT solution (1 mg/mL final concentration) at 37° C for 4 h. The formazan crystals were dissolved using dimethyl sulfoxide (DMSO, 150 μL/well, Beyotime). The optical density was read at 570 nm using a microplate reader (Molecular Devices). Cell viability was quantified as the percentage of MTT reduction compared to the control conditions without any treatments.

### Real time-quantitative PCR (RT-qPCR) analysis

RT-qPCR analysis was conducted as previously indicated [62]. In brief, total RNA was extracted from renal tissues or cells using Trizol reagent (Invitrogen, USA) following the manufacturer’s instructions. Then, the obtained total RNA was reverse transcribed using M-MLV-RT system (Promega, USA), which was performed at 42° C for 1 h and terminated through deactivation of the enzyme at 70° C for 10 min. Subsequently, PCR was conducted with SYBR Green (Bio-Rad, USA) on an ABI PRISM 7900HT detection system (Applied Biosystems, USA). All primer sequences used in the study were obtained from Invitrogen Corporation or Generay Biotech (Shanghai, China), and were listed in Supplementary Tables 1, 2. The quantification of each gene was analyzed according to the 2^-ΔΔCt^ expressions. ΔΔCt represents the relative change in the differences between the control and treatment groups. Expression of each mRNA was normalized with GAPDH mRNA.

### Western blot analysis

Nuclear and Cytoplasmic Protein Extraction kit (KeyGEN BioTECH, Nanjing, China) was used to extract nuclear protein and cytoplastic protein according to the manufacturer’s instructions. Kidney tissue samples and cells were homogenized using 10% (wt/vol) hypotonic buffer (5 μg/ml soybean trypsin inhibitor, 1 mM EDTA, 4 mM benzamidine, 1 mM Pefabloc SC, 25 mM Tris-HCl, 5 μg/ml leupeptin, 50 μg/ml aprotinin, pH 8.0) to yield a homogenate. The final supernatants were then obtained via centrifugation at 12,000 rpm for 15 min at 4° C. Next, BCA protein analysis kit (Thermo Fisher Scientific, USA) was used to determine the protein concentration according to the protocols provided by the manufacturer. 40-50 μg of total protein was subjected to 10% or 12% SDS-polyacrylamide gels, and electrophoretically transferred to polyvinylidene difluoride (PVDF) membranes (Millipore Corporation, USA), followed by incubation with primary antibodies (Supplementary Table 3) overnight at 4° C. Next, these membranes were incubated with HRP-conjugated secondary antibodies (Supplementary Table 3) for 1 h at room temperature. Finally, the signal was detected with ECL Detection system (Thermo Fisher Scientific). Each protein expression was quantified using the Image Lab Software (Version 1.4.2b, National Institutes of Health, USA), normalized to GAPDH or Lamin B and expressed as a fold of change.

### Biochemical analysis *in vitro* or *in vivo*

Serum creatinine, blood urea nitrogen (BUN), and urinary albumin levels were assessed using an AU680 automated chemistry analyzer (Beckman Coulter, Inc., USA). Insulin enzyme-linked immunosorbent assay (ELISA) kit (Sigma Aldrich) was used to determine serum insulin levels according to the supplier’s protocols. The serum triglyceride (TG), total cholesterol (TC) and low-density lipoprotein cholesterol (LDL-C) were measured using commercial kits also purchased from Nanjing Jiancheng Bioengineering Institute (Nanjing, China) following the manufacturer’s protocols. The contents of malondialdehyde (MDA) and superoxide dismutase (SOD) were evaluated using commercial kits obtained from Nanjing Jiancheng Bioengineering Institute in accordance with the manufacturer’s instructions. The blood glucose levels were determined with an o-toluidine reagent (Sigma Aldrich, USA). H_2_O_2_ contents in cells or renal samples were measured using commercial kit purchased from Solarbio Life Science (#BC3595) following the instructions provided by the manufacturer. As for hepatic toxicity analysis, serum alanine aminotransferase (ALT), aspartate aminotransferase (AST) and alkaline phosphatase (ALP) were measured using commercial kits (Nanjing Jiancheng Bioengineering Institute) according to the manufacturer’s instructions.

### Oral glucose tolerance tests (OGTT) and insulin tolerance tests (ITT) assays

OGTT and ITT were performed to calculate the insulin resistance in Fru-fed mice. After fasting for 8 h, mice were orally treated with glucose (2 g/kg body weight). Immediately after glucose administration, the blood samples were harvested from the tail vein at the shown time (0, 15, 30, 60 and 120 min). Subsequently, the blood glucose levels were measured with the o-toluidine reagent (Sigma Aldrich). As for ITT analysis, the mice were fasted for 8 h before i.p. injection with insulin (1 U/kg body weight, Sigma Aldrich). Finally, the blood glucose levels were calculated at the indicated time post insulin injection.

### ROS measurements *in vitro* or *in vivo*

Dichlorofluorescein-diacetate (DCFH-DA, KeyGEN BioTECH) was used for the measurements of cellular ROS production. In brief, after treatments, DCFH-DA (10.0 μM, 1.5 mL) was added to each well and incubated for 30 min at 37° C after removing the medium. The samples were finally observed under a fluorescence microscopy (Olympus, Japan) and the fold change of relative ROS levels was quantified.

Dihydroethidium (DHE) (Invitrogen, USA) was used to measure ROS levels in renal samples of mice. In brief, the kidney was fixed in 4% paraformaldehyde for 24 h, dehydrated with 10% and 5% sucrose, embedded and cut into 8-μm sections. After washing with PBS, the sections were placed into 2 μmol/L of DHE dye, followed by incubation at 37° C for 30 min. After washing, the sections were analyzed and quantified by Image J software.

### Animals and treatments

Male C57BL/6 mice (6-7 weeks of age, weighing 18-20 g) were purchased from the Beijing Vital River Laboratory Animal Technology Co., Ltd (Beijing, China). All animal treatment procedures were approved by the Institutional Animal Care and Use Committee in Chongqing Key Laboratory of Medicinal Resources in the Three Gorges Reservoir Region, School of Biological and Chemical Engineering, Chongqing University of Education, and were performed in accordance with the Guide for the Care and Use of Laboratory Animals, issued by the National Institutes of Health in 1996. The protocols performed in the study were in accordance with the Regulations of Experimental Animal Administration issued by the Ministry of Science and Technology of the People’s Republic of China (http://www.most.gov.cn). All mice were housed in a constant temperature (25 ± 1° C), humidity (50 ± 5%) and specific pathogen-free (SPF) controlled environment under a 12-h light: 12-h dark cycle with free access to food and water. After adaption for 7 days, mice were randomly divided into 5 groups as follows: the control group (Con), the control group plus higher concentration of CA (1% CA; CAH), Fru group, Fru plus lower concentration of CA group (0.5% CA; Fru+CAL), and Fru plus higher concentration of CA group (1% CA; Fru+CAH). Fru group of mice was fed with water containing 30% (w/v) of Fru. The concentration of Fru solution used in this study was decided based on previous reports [63, 64]. Fru-challenged mice were simultaneously supplemented with 0.5% or 1% CA (HPLC ≥ 95%, Sensient Technologies) dissolved in 30% Fru solution. The precise dosage of CA (0.75 g/kg/daily and 1.5 g/kg/daily, respectively) was evaluated for each mouse per day according to the body weight. The body weight of mice was measured every week. After 16 weeks, the mice were sacrificed, and the blood (0.6-1.0 ml) was harvested from the eyeball of each animal. The kidney samples of mice were isolated and measured, and then were stored at -80° C for further assays. In addition, major organs including liver, spleen, lung and heart were collected for toxicity analysis. Prior to sacrifice, each mouse was placed in a metabolic cage to collect 24 h urine, which was centrifuged at 4° C (3000 × g for 10 min) to remove particulate contaminants and stored at -80° C for albumin assessment.

### Histological analysis

The major organs including kidney, heart, liver, spleen and lung were isolated from each group of mice, fixed in 4% paraformaldehyde, implanted in paraffin, and then sectioned transversely at 3-μm thickness. Then, the renal sections were subjected to hematoxylin and eosin (H&E), periodic acid-Schiff (PAS), Sirius Red and Masson’s trichrome staining to evaluate the histological alterations of renal samples. The glomerular area was quantified as previously described [65]. As for immunohistological staining, the paraffin sections were dewaxed, washed in phosphate buffered saline (PBS), and then incubated in preheated 10 mmol/L sodium citrate buffer at 94° C for 15 min. All sections were rinsed and blocked using 10% normal goat serum for 30 min and then incubated with primary antibodies including 8-hydroxydeoxyguanosine (8-OHdG; diluted at 1:100, ab48508, Abcam, USA) and 4-hydroxynonenal (4-HNE; diluted at 1:100, ab48506, Abcam) overnight at 4° C, followed by incubation with secondary antibody (ab6728, Abcam) for 40 min at 37° C. After washing with PBS, the slides were developed with 3,3’-diaminobenzidine (DAB, Sigma Aldrich) containing 0.03% hydrogen peroxide. Finally, these hepatic sections were counterstained with hematoxylin for 1 min. The positive areas were observed and captured under a light microscope. All histological measurements were analyzed by 3 investigators blinded to the treatment procedures.

### Statistical analysis

Data are shown as means ± standard error of the mean (SEM) and analyzed by the Student’s t-test or one-way or two-way ANOVA followed by Bonferroni post hoc tests in which appropriate P values less 0.05 are considered statistically significant. Results were evaluated using GraphPad Prism 6 (GraphPad Software, USA). Animal feeding, treatment and histological analysis were performed in a single-blinded fashion. No samples or animals were excluded from the analysis.

## Supplementary Material

Supplementary Figures

Supplementary Tables
