# Supplementary material for: Carminic acid supplementation protects against fructose-induced kidney injury mainly through suppressing inflammation and oxidative stress via improving Nrf-2 signaling
Source: Aging (Albany NY). 2021 Apr 4;13(7):10326–53. doi: 10.18632/aging.202794 (PMC8064181; doi:10.18632/aging.202794)
Supplement: Supplementary Figures [file aging-13-202794-s001.pdf]

SUPPLEMENTARY FIGURES

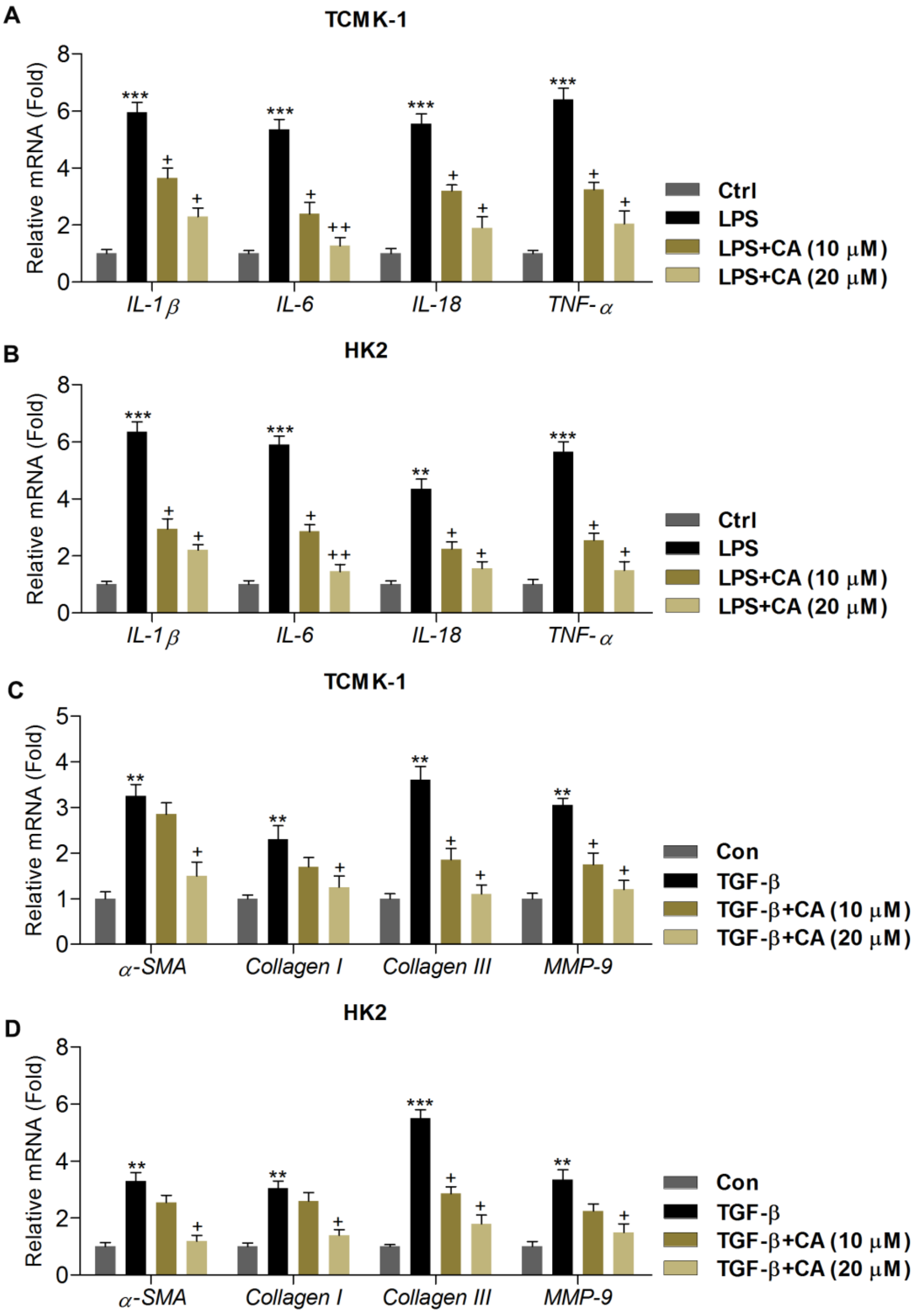

Supplementary Figure 1. Carminic acid exhibits anti-inflammatory and anti-fibrotic effects *in vitro*. (**A**) TCMK-1 and (**B**) HK2 cells were treated with 100 ng/ml of LPS for 24 h in the absence or presence of CA (10 and 20 μM), and were then collected for RT-qPCR analysis of IL-1β, IL-6, IL-18 and TNF-α. (**C**) TCMK-1 and (**D**) HK2 cells were exposed to 10 ng/ml of TGF-β for 24 h with or without CA (10 and 20 μM). Then, all cells were harvested for RT-qPCR analysis of fibrotic genes including α-SMA, Collagen I, Collage III and MMP-9 in cells as treated. The results are expressed as the means ± SEM. n = 4 in each group. $^{**}P < 0.01$ and $^{***}P < 0.001$ compared with the Ctrl group; $^{+}P < 0.05$ and $^{++}P < 0.01$ compared with the LPS or TGF-β group.

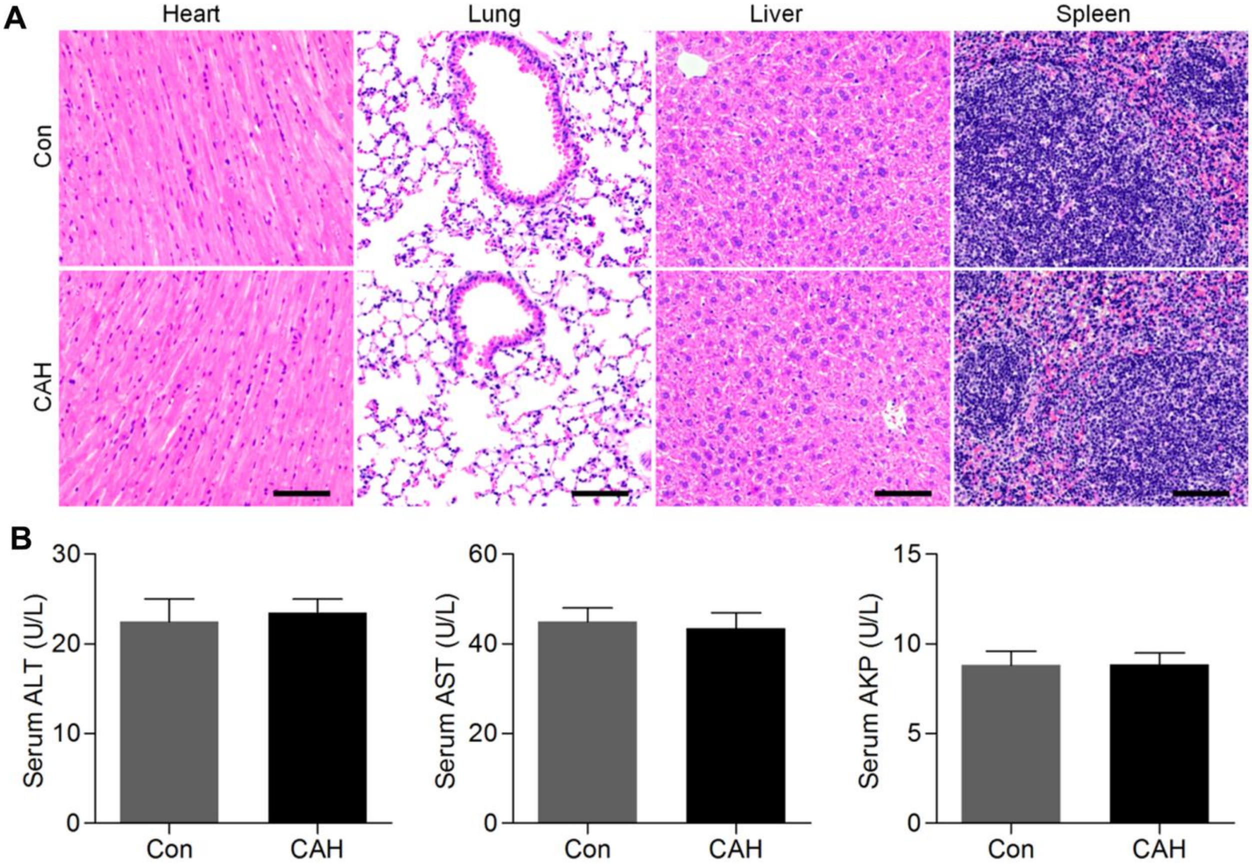

Supplementary Figure 2. Toxicity analysis of carminic acid *in vivo.* (**A**) H&E staining for heart, lung, liver and spleen in mice treated with or without carminic acid. (**B**) Assessments of serum ALT, AST and AKP in mice from the indicated groups. The results are expressed as the means ± SEM. n = 6 in each group.
