# Supplementary material for: Carminic acid supplementation protects against fructose-induced kidney injury mainly through suppressing inflammation and oxidative stress via improving Nrf-2 signaling
Source: Aging (Albany NY). 2021 Apr 4;13(7):10326–53. doi: 10.18632/aging.202794 (PMC8064181; doi:10.18632/aging.202794)
Supplement: Supplementary Tables [file aging-13-202794-s002.pdf]

SUPPLEMENTARY TABLES

Supplementary Table 1. Primer sequences of RT-qPCR test for mouse.

Items	Primer (5'→3')
IL-4 (forward)	TCTCTCTGAAGGACTCTGGCT
IL-4 (reverse)	CTTTGCCCACGGACACAACT
IL-1β (forward)	GCCACCTTTTGACAGTGATGAG
IL-1β (reverse)	AGTGATACTGCCTGCCTGAAG
TNF-α (forward)	ACCTGGCCTCTCTACCTTGT
TNF-α (reverse)	CCCGTAGGGCGATTACAGTC
IL-6 (forward)	CAACGATGATGCACTTGCAGA
IL-6 (reverse)	TCTCTCTGAAGGACTCTGGCT
IL-18 (forward)	TTGATCCCACTTCGTGCTTTCA
IL-18 (reverse)	CCTTTCCTCTTCCCGAAGCTGT
TGF-β1 (forward)	TGACGTCACTGGAGTTGTACGG
TGF-β1 (reverse)	GGTTCATGTCATGGATGGATGGTGC
TIMP1 (forward)	CCCTTTGCATCTCTGGCATC
TIMP1 (reverse)	GCATTTCCCACAGCCTTGAA
MIP-1α (forward)	CCCGACTGCCTGCTGCTTCTCC
MIP-1α (reverse)	CAAAGGCTGCTGGTCTCAAA
MCP-1 (forward)	CACCTGCTGCTACTCATTCAC
MCP-1 (reverse)	CTGTCACACTGGTCACTCCTAC
CXCL1 (forward)	ACCTATGGCCCTGACATCATCAC
CXCL1 (reverse)	CTTGCCAGCCCTCAGAATCAC
SOD1 (forward)	AAGGCCTGCATGGATTCCA
SOD1 (reverse)	AAGGCCTGCATGGATTCCA
SOD2 (forward)	AAGGCCTGCATGGATTCCA
SOD2 (reverse)	AAGGCCTGCATGGATTCCA
HO-1 (forward)	CGTGCTCGAATGAACACTCT
HO-1 (reverse)	GGAAGCTGAGAGTGAGGACC
Nrf-2 (forward)	ACCTCCCTGTTGATGACTT
Nrf-2 (reverse)	GGCGACTTTATTCTTACCTCT
GCLM (forward)	ATGGAGTTCCCAAATCAGCC
GCLM (reverse)	ATTGGGTTTTACCTGTGCCC
GCLC (forward)	CCATCACTTCATTCCCCAGA
GCLC (reverse)	GATGCCGGATGTTTCTTGTT
Keap-1 (forward)	CAACTTCGCGGAGCAGATCG
Keap-1 (reverse)	AGCTGGCAGTGTGACAGGTT
iNOS (forward)	GGAAGAAACAAAACCCTCT
iNOS (reverse)	CCTGGCAGTCACAGTCATA
NQO-1 (forward)	CAGCCAATCAGCGTTCGGTA
NQO-1 (reverse)	TTGCTGTTGAGGTCGCAGGAG
$Gp91^{phox}$ (forward)	TTGGGTCAGCACTGGCTCTG
$Gp91^{phox}$ (reverse)	TGGCGGTGTGCAGTGCTATC
$p22^{phox}$ (forward)	AACGAGCAGGCGCTGGCGTCCG
$p22^{phox}$ (reverse)	CACAGTGGTATTTCGGCGCC
$p47^{phox}$ (forward)	CCAGCACTATGTGTACATGT
$p47^{phox}$ (reverse)	AAGGAGATGTTCCCCATTGA
XO (forward)	AAAGGACCAGACGATTGCTCC

XO (reverse)	TCACACGTTCCCCTTCAAAAC
α-SMA (forward)	TTCCTTCGTGACTACTGCCG
α-SMA (reverse)	TATAGGTGGTTTCGTGGATGCC
Collagen I (forward)	AGGCTTCAGTGGTTTGGATG
Collagen I (reverse)	CACCAACAGCACCATCGTTA
Collagen III (forward)	CCCAACCCAGAGATCCCATT
Collagen III (reverse)	GAAGCACAGGAGCAGGTGTAGA
MMP-9 (forward)	CGTCATTCGCGTGGATAAGG
MMP-9 (reverse)	TTTGGAAACTCACACGCCAG
PEPCK (forward)	TGCCCCAGGCAGTGAGGAAGTT
PEPCK (reverse)	GTCAGTGAGAGCCAGCCAACAGT
G6PC (forward)	TCTGTCCCGGATCTACCTTG
G6PC (reverse)	GCTGGCAAAGGGTGTAGTGT
FBP1 (forward)	GCATCGCACAGCTCTATGGT
FBP1 (reverse)	ACAGGTAGCGTAGGACGACT
SCD1 (forward)	CCTACGACAAGAACATTCAATCCC
SCD1 (reverse)	ACTCACTGGCAGAGTAGTCGAA
PPARγ (forward)	ATTCTGGCCCACCAACTTCGG
PPARγ (reverse)	TGGAAGCCTGATGCTTTATCCCCA
FAS (forward)	CTGCGGAAACTTCAGGAAATG
FAS (reverse)	GGTTCGGAATGCTATCCAGG
CPT1α (forward)	CTCAGTGGGAGCGACTCTTCA
CPT1α (reverse)	GGCCTCTGTGGTACACGACAA
PPARα (forward)	CAAGGCCTCAGGGTACCACT
PPARα (reverse)	TTGCAGCTCCGATCACACTT
GAPDH (forward)	GGTGAAGGTCGGTGTGAACG
GAPDH (reverse)	CCCGTAGGGCGATTACAGTC

Supplementary Table 2. Primer sequences of RT-qPCR test for human.

Items	Primer (5’→3’)
IL-4 (forward)	TTTGAACCAGGTCACAGA
IL-4 (reverse)	GACCGCTGACACCTCTAC
IL-1β (forward)	CCTGCGTGTTGAAAGATGATAA
IL-1β (reverse)	CTGCTTGAGAGGTGCTGATGTA
TNF-α (forward)	CCTCTCTCTAATCAGCCCTCTG
TNF-α (reverse)	GAGGACCTGGGAGTAGATGAG
IL-6 (forward)	CCAGGAGCCCAGCTATGAAC
IL-6 (reverse)	CCCAGGGAGAAGGCAACTG
IL-18 (forward)	AGAGCGCAATGGTG
IL-18 (reverse)	GACGCATGCCCTCAA
TIMP1 (forward)	GGGCTTCACCAAGACCTACA
TIMP1 (reverse)	TGCAGGGGATGGATAAACA
MIP-1α (forward)	CAGCGAGTACCAGTCCCTTTT
MIP-1α (reverse)	CCTCGCTGCCTCCAAGA
MCP-1 (forward)	GTCTCTGCCGCCCTTCTG
MCP-1 (reverse)	ACTTGCTGCTGGTGATTCTTCT
CXCL1 (forward)	GTGGAAGGTGCTTGCACACCAGG
CXCL1 (reverse)	GGAGCAGCAGTGCCACTCGCAGG
SOD1 (forward)	TGGATCTGCCAACTACTCCC

SOD1 (reverse)	CGTAGCCGAAGAAACCTCAT
SOD2 (forward)	CCATTTTCTGGACAAACCTGA
SOD2 (reverse)	GACCCAAAGTCACGCTTGATA
HO-1 (forward)	GAGAATGCTGAGTTCATG
HO-1 (reverse)	ATGTTGAGCAGGAAGGC
Nrf-2 (forward)	TTCAGCAGCATCCTCTCCACAG
Nrf-2 (reverse)	GCATGCTGTTGCTGATACTGG
GCLM (forward)	GCTTCTTGGAAACTTGCTTCA
GCLM (reverse)	CTGTGTGATGCCACCAGATT
GCLC (forward)	TGTTTCCTGGACTGATCCCAA
GCLC (reverse)	TGCGATAAACTCCCTCATCCAT
Keap-1 (forward)	CACAGCAGCGTGGAGAGA
Keap-1 (reverse)	CAACATTGGCGCGACTAGA
iNOS (forward)	CGGTGCTGTATTTCCTTACGAGGCGAAGAA
iNOS (reverse)	GGTGCTGCTTGTTAGGAGGTCAAGTAAAGG
NQO-1 (forward)	ACGCCCGAATTCAAATCCT
NQO-1 (reverse)	CCTGCCTGGAAGTTTAGGTC
Gp91^{phox} (forward)	AAGATAGCGGTTGATGGGC
Gp91^{phox} (reverse)	TTGAGAATGGATGCGAAGG
p22^{phox} (forward)	CGCTTCACCCAGTGGTACTT
p22^{phox} (reverse)	CAGCCGCCAGTAGGTAGATG
p47^{phox} (forward)	GGGTGATGTGGTGGATGTCG
p47^{phox} (reverse)	TGTCCTTTGAGTCAGGGCTC
XO (forward)	GCATATCATTGGTGCTGTGG
XO (reverse)	GGTCCCCTTTCTCGATCTTC
Pepck (forward)	AAGTATGACAACTGCTGGTTGGC
Pepck (reverse)	ATAACCGTCTTGCTTTCGATCCT
	Proteinkinase ATAACCGTCTTGCTTTCGATCCT
G6Pc (forward)	CCCCTGATAAAGCAGTTCCCT
G6Pc (reverse)	ATACACCTGCTGTGCCCATG
	Nuclear ATACACCTGCTGTGCCCATG
	Nuclear ATACACCTGCTGTGCCCATG
	Nuclear ATACACCTGCTGTGCCCATG
FBP1 (forward)	GATTGCCTTGTGTCCGTTG
FBP1 (reverse)	TGCCATACAGTGCGTAGCC
α-SMA (forward)	TCCGGAGCGCAAATACTCTG
α-SMA (reverse)	CCCGGCTTCATCGTATTCCT
Collagen I (forward)	GTGCTCCTGGTATTGCTGGT
Collagen I (reverse)	TCCTTGAACACCAACAGGGC
Collagen III (forward)	TTCGACTTCTCTCCAGCCGA
Collagen III (reverse)	TCCACTGGCCTGATCCATGT
MMP-9 (forward)	CACTGTCCACCCCTCAGAGC
MMP-9 (reverse)	GCCACTTGTCGGCGATAAGC
GAPDH (forward)	GGAGTCAACGGATTTGGTC
GAPDH (reverse)	GGCAACAATATCCACTTTACC

Supplementary Table 3. Primary and secondary antibodies for western blot analysis.

Name	Catalog	Dilutions	Vendor
Phospho-NF-κB	ab86299	1:1000	Abcam
NF-κB	ab76311	1:1000	Abcam
Phospho-IκB-α	#2859	1:1000	Cell Signaling Technology
IκB-α	#4814	1:1000	Cell Signaling Technology
Nrf-2	ab31163	1:1000	Abcam
Phospho-IKKα	ab38515	1:1000	Abcam
IKKα	ab32041	1:1000	Abcam
Keap-1	ab150654	1:1000	Abcam
Phospho-JNK	9255	1:1000	Cell Signaling Technology
JNK	9252	1:1000	Cell Signaling Technology
Podocin	ab50339	1:1000	Abcam
Lamin B	ab133741	1:1000	Abcam
AMPKα	ab80039	1:1000	Abcam
Phospho-AMPKα	ab23875	1:1000	Abcam
Rabbit IgG	ab6721	1:4000	Abcam
Mouse IgG	ab6728	1:4000	Abcam
GAPDH	ab8245	1:1000	Abcam
